# Serum ferritin levels and irregular use of iron chelators predict liver iron load in patients with major beta thalassemia: a cross-sectional study

**DOI:** 10.3325/cmj.2019.60.405

**Published:** 2019-10

**Authors:** Soheila Sobhani, Farzaneh Rahmani, Maryam Rahmani, Marzieh Askari, Farzad Kompani

**Affiliations:** 1 Students’ Scientific Research Center, Tehran University of Medical Sciences, Tehran, Iran; 2 NeuroImaging Network (NIN), Universal Scientific Education and Research Network (USERN), Tehran, Iran; 3 Devision of Hematology and Oncology, Children’s Medical Center, Pediatric Center of Excellence, Tehran University of Medical Sciences, Tehran, Iran

## Abstract

**Aim:**

To determine whether serum ferritin, liver transaminases, and regularity and type of iron chelation protocol can be used to predict liver iron load as assessed by T2* magnetic resonance imaging (MRI) in patients with beta thalassemia major (TM).

**Methods:**

This cross-sectional study, conducted from March 1, 2014 to March 1, 2015, involved 90 patients with beta TM on regular packed red blood cell transfusion. Liver and cardiac iron load were evaluated with T2* MRI. Compliance with iron-chelating agents, deferoxamine or deferasirox, and regularity of their use, as well as serum ferritin and liver transaminase levels were assessed.

**Results:**

Patients with high serum ferritin were 2.068 times (95% confidence interval 1.26-3.37) more likely to have higher liver or cardiac iron load. High serum aspartate aminotransferases and irregular use of iron chelating agents, but not their type, predicted higher cardiac iron load. In a multiple regression model, serum ferritin level was the only significant predictor of liver and myocardial iron load.

**Conclusions:**

Higher serum ferritin strongly predicted the severity of cardiac and liver iron load. Irregular use of chelator drugs was associated with a higher risk of cardiac and liver iron load, regardless of the type of chelating agent.

Sobhani et al: Serum ferritin levels and irregular use of iron chelators predict liver iron load in patients with major beta thalassemia

## 

Excess iron load, resulting from increased gastrointestinal absorption and repeated red blood cell transfusions, in patients with thalassemia heralds a wide range of complications, including arrhythmia and congestive heart failure (1). Almost 50% of patients with beta-thalassemia major (TM), a transfusion-dependent form of thalassemia syndromes, are affected by myocardial siderosis before the age of 35 and are at risk of congestive heart failure (2) as a major cause of morbidity (3). Also, it is important to point out that in patients with beta TM, transfusion-dependent complications comprise the highest share of economic (4) and global burden (5).

Two main predictors of survival in TM patients are iron load in organs and adherence to chelating drugs (6). Indeed, persistent serum ferritin above 2500 ng/mL and liver iron concentration (LIC) above 15 mg/g dry weight can significantly predict 10-year survival of patients with TM (7). Currently there are three available iron-chelating drugs: deferoxamine (DFO) as the first available agent, deferiprone (DFP), and deferasirox (DFX) (6), which are shown to be equally effective in preventing complications (8,9).

Magnetic resonance imaging (MRI) is a non-invasive and rapid method of measurement of tissue iron in the cardiac muscle and liver. Its results are presented in terms of R2, R2*, T2, and T2* (10). The T2 star (T2*) is inherently similar to T2 signal in MRI, yet it gives a more realistic estimate of signal decoy due to intrinsic tissue properties, such as excess iron load (11). T2* value of less than 20 ms is a strong predictor of higher risks for arrhythmia and/or congestive heart failure (CHF) (12). Liver and cardiac iron load assessed by MRI T2*, together with iron chelating therapy, can be used as valuable indicators of transfusion-dependent side effects, as certified by the notable decrease in the overall mortality rate of beta-TM patients after the emergence of cardiac T2* MRI in 1999 (13).

Despite reliable results obtained by T2*MRI (14), especially for myocardial iron load in predicting heart failure (15), there are debates about the relevance of liver and cardiac iron concentrations for the overall prognosis. Current guidelines recommend the liver and cardiac T2*MRI to be performed annually in all patients older than 6 years with ferritin above 1000 ng/mL, relying on the fact that serum ferritin is the marker of choice to monitor total body iron storages (16). A part of the inter-individual heterogeneity in total body iron load is predicted by iron chelation protocol (17-19).

Pursuing the idea that serum markers can be used as a proxy to total body and liver iron load, we aimed to develop a model to predict cardiac and liver iron load using ferritin, liver transaminases, and regularity and type of iron chelation protocol. We hypothesize that liver transaminases, serum ferritin, and dosage of iron chelating treatment can add to the predictive value of iron load in T2*MRI.

The aims of the current study were to assess: 1) whether serum ferritin level can independently predict hepatic or cardiac iron load, 2) whether the dosage or type of iron chelating agent is able to estimate the degree of hepatic or cardiac iron load, 3) whether the severity of hepatic or cardiac iron load can be predicted by serum AST or ALT.

## Material and methods

### Study design

This single-center cross-sectional study was conducted from March 1, 2014 to March 1, 2015 in the Thalassemia Clinic of Children’s Medical Center, Pediatric Center of Excellence in Tehran, Iran.

The sample size was calculated based on the formula for cross-sectional studies (20):


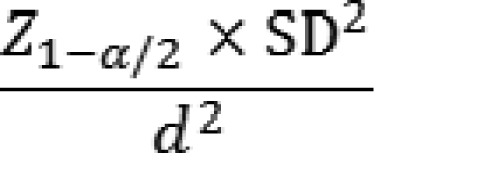


Adopting 0.05 as the significance level (Z_α/2_ = 1.96), standard deviation from the study by Majd et al (21), and d of 10 ng/dL for ferritin and 2 milliseconds for cardiac iron load, we calculated that the sample size for the estimation of serum ferritin levels should be 100 and for myocardial and liver iron load estimation it should be 88.

### Participants’ demographic data

Out of 154 patients with beta-TM treated at the Thalassemia Clinic, 138 accepted to participate in the study. The diagnosis of beta-TM was based on patient’s clinical history, complete blood count, and hemoglobin electrophoresis performed at the Children’s Medical Center laboratory. Medical records of all patients were reviewed. In patients who did not have serum viral markers obtained in the period of at most three months before the study, viral marker examination was requested. Three patients were excluded due to co-morbid hepatitis C infection. Ten more patients were excluded due to abnormal liver ultrasonography and 35 patients due to missing results from T2* MRI. Finally, the study recruited 90 patients (51 or 56.7% female) with beta-TM and regular packed red blood cell (RBC) transfusion and a mean age of 20.5 ± 7.6 years (range 7-25).

### Transfusion history, type, and dosage of iron-chelating agents

All patients had received at least 8 regular packed RBC transfusions per year for the preceding 3 years. Transfusion duration was calculated from the date of the first registration at the clinic and imaging/sampling date. At the time of the sampling, all patients were receiving iron chelators, including DFO, DFX, or both.

Iron chelation therapy in all patients had been started with DFO after at least 10 transfusions or after serum ferritin increased to above 1000 μg/L, whichever came first (22). Treatment was started at 20 mg/kg administered in an 8-hour transfusion 5 times a week in children under the age of 16, with the goal to maintain serum ferritin below 2000 μg/L, or better below 1000 μg/L, without increasing the dose over 40 mg/kg (22). For children under the age of 2 years, DFX was started as first-line therapy with 10 mg/kg/day tablets, and titrated to obtain serum ferritin below 1000 μ/L. DFO and DFX dose were titrated based on serum ferritin levels obtained three-monthly and annual liver and heart T2* MRI. No patient had been registered after 16 years of age.

Compliance with DFO was calculated as the percentage of completed infusions by the MP Thalapump 20 (Micrel Medical Devices, Athens, Greece) specified for each patient, as described before (23). Compliance with DFX was assessed with a self-report questionnaire, either filled out by the patient or a caregiver/parent if the patient was not cognitively competent. Adequate dosing of DFO was defined as a daily dose of 20-40 mg/kg for children and 50-60 mg/kg for adults (22), subcutaneously injected 5-6 times a week, started before the age of six years. Adequate dosing of DFX was defined as a daily oral intake of 20-40 mg/kg of dispersible tablets, started before the age of six years (24). The cut-off levels for regular iron chelators adherence were adopted from the study by Cohen et al (25). Patients were, therefore, categorized as “irregular users” if in three months of average follow-up they had received less than 50 mg/kg/day (20 mg/kg/day in children) of DFO or less than 30 mg/kg/day of DFX. Importantly, no change in type or dosage of medications had occurred in the 6 months preceding the date of imaging in any of the patients.

### T2* magnetic resonance imaging (MRI)

All Thalassemia Clinic patients undergo annual examination for cardiac and liver iron load by liver and heart T2* MRI (Siemens, Avanto, 1.5 Tesla, Munich, Germany) at the Pardis Noor referral center of Iranian Red Crescent Organization in Tehran. The imaging was scheduled for at least 5 days of the last transfusion.

Similar to the T2 signal in MRI, the T2* signal measures the time constant of magnetization decoy, ie, relaxation time. Due to its paramagnetic properties, excess iron accumulation in the tissue shortens the relaxation time, hence accelerating the decrease in signal intensity (26). Susceptibility-induced distortions in the magnetic field result in a faster signal decoy compared to the true relaxation time (T2) predicted for the tissue. T2* hence measures the effective “observed” T2, yielding a lower value compared with the predicted T2 (11). T2* has been shown in patients with mild to moderate iron overload to be a reliable indicator of LIC (26,27) and cardiac iron load (28). T2* less than 20 ms indicates a high LIC, while T2* of less than 10 ms indicates a severely high LIC. Compared with LIC, T2* generally less accurately estimates cardiac iron concentration (22).

The severity of iron overload in the heart and liver was determined from the patients’ most recent T2* liver and heart MR images. According to the manufacturer’s guideline, liver iron load was considered normal if hepatic T2* values were above 6.3 ms, mild if they ranged from 2.8-6.3 ms, moderate if they ranged from 1.4-2.7 ms, and severe if they were below 1.4 ms. Also, a T2* signal above 20 ms indicated normal cardiac iron load, a signal from 14-20 ms indicated mild iron load, a signal from 10-14 ms indicated moderate iron load, while a signal lower than 10 ms indicated severe iron.

### Serum measurements, echocardiography, and liver ultrasonography

Serum aspartate aminotransferase (AST), alanine aminotransferase (ALT), and serum ferritin levels were measured at the time of MRI imaging.

Since fatty liver and liver heterogeneity are common confounding factors in the measurement of liver iron load, patients were also scheduled for abdominal ultrasonography (GE VIVID 3, GE Medical Systems, Chicago, IL, USA, probe 3 MHz) by an expert radiologist, to assess whether they have co-existing fatty liver or active hepatitis. All patients also underwent echocardiography (GE VIVID 3, probe 3 MHz). Hence, ten patients were excluded due to abnormal liver ultrasonography findings.

Importantly, laboratory and imaging procedures were performed at no expense for the patients. Before being asked to sign informed consent forms, patients or their legal guardians were informed about the aims of the study and assured that their anonymity would be respected. The study was approved by the review board of Children’s Medical Center and Iranian Red Crescent Organization.

### Statistical analysis

The Kolmogorov-Smirnov test was used to assess the normality of distribution of age, sex, serum AST, serum ALT, and ferritin levels. To assess if AST, ALT, ferritin, regularity of iron chelator use, and age were significantly associated with the stages of liver and cardiac iron load we used the χ^2^ test or Fisher exact test, where applicable, and calculated the odds ratios (OR). Age was entered into the non-parametric comparison with use of Mann-Whitney U tests with previous dichotomous variables. Finally, the variables that were significant in previous analyses were entered into a multiple linear regression analysis to assess if they predicted liver or cardiac iron load independently of other variables. We tested multivariate normality through normal Q-Q plots for each significant variable. We entered the variables in a step-wise manner, meaning that the next variable was entered if the probability of F remained significant at 0.05 levels. Collinearity was assessed with use of Durbin-Watson test and homoscedasticity of all significant and non-significant variables by *post-hoc* plotting of square residues against predicted values. The level of significance was set at less than 0.05. Statistical analyses were performed with SPSS, version 23 (IBM, Armonk, NY, USA).

## Results

The study involved 90 patients (51 or 56.7% female), with the mean age of 20.5 ± 7.6 years. Most patients were older than 20 years (n = 54, 60%), 32 patients were between 10-20 years old, and only 4 patients were younger than 10 years. Most patients (n = 84, 93.3%) received RBC transfusion for 5-20 years, one patient for more than 20 years, and 5 patients (5.6%) for less than 5 years. Fifty-two patients received DFO (Desferal, Novartis, Basel, Switzerland) (57.7%), 29 patients DFX (Exjade, Novartis) (32.2%), and 9 patients (10%) received both. Most patients used iron chelators regularly (n = 61, 67.8%). The cut-off for liver aminotransferases was 40 IU/L (in patients older than 14 years and age-specific cut-off for patients younger than 14 years). According to this cut-off, 79.9% and 86.7% of patients had AST and ALT levels within the reference range, respectively. According to the ferritin cut-off of less than 2000 ng/mL, the patients were categorized to “normal/low” or “abnormal/high” ferritin groups (Table 1). Based on the cut-off values described in the methods section, 36 (40%) patients had mild liver iron load, 27 (30%) patients had moderate iron load, and 4 (4.4%) patients had severe iron load (Table 1). Most patients had normal (n = 55, 61%) or mild (n = 20, 22.2%) cardiac iron load (Table 1).

**Table 1:** The proportion of patients with normal/abnormal aspartate aminotransferase, alanine aminotransferase, and ferritin levels and in each group based on T2*magnetic resonance imaging of liver and heart iron load

Parameter	No. (%) of patients
Serum aspartate aminotransferase (IU/dL)	
low to normal	71 (78.9)
high/abnormal	19 (21.1)
Serum alanine aminotransferase (IU/dL)	
low to normal	78 (86.7)
high/abnormal	12 (13.3)
Serum ferritin (ng/dL)	
low to normal	51 (56.7)
high/abnormal	39 (43.3)
Liver iron load*	
normal	23 (25.6)
mild	36 (40)
moderate	27 (40)
severe	4 (4.4)
Myocardial iron load*	
normal	55 (61.1)
mild	20 (21.2)
moderate	7 (7.8)
severe	8 (8.9)

*Liver and myocardial iron load are based on T2*MRI values expressed in milliseconds.

There was no difference in liver or heart iron load between male and female patients (Table 2). Also, the cross-tabulation of age groups with different grades of liver and heart iron load revealed no significant results (Table 2). Significantly more men (46.1% vs 25.5%) than women had moderate to high liver iron load (*P* = 0.032, chi square test). Hence, women were 1.53 times less likely to have moderate to high liver iron load (odds ratio [OR] 1.53, 95% confidence interval [CI] 1.08-2.42, *P* = 0.041). No sex difference in cardiac iron load was observed.

**Table 2:** Liver and heart iron load according to sex, and serum aspartate aminotransferase (AST), alanine aminotransferase (ALT), and ferritin levels

	No. (%) of patients with																				
Parameter	liver iron load											heart iron load									
	normal		mild		moderate			severe		P*		normal		mild		moderate		severe		P*	
Sex																				
male		6 (15.4)		15 (38.5)		17 (43.6)	1 (2.6)		0.032		31 (60.8)		12 (23.5)		4 (7.8)		4 (7.8)		0.966	
female		17 (33.3)		21 (41.2)		10 (19.6)	3 (5.9)				24 (61.5)		8 (20.5)		3 (7.7)		4 (10.3)			
Serum AST																				
low		18 (25.4)		33 (46.5)		18 (25.4)	2 (2.8)		0.107		47 (66.2)		15 (21.1)		5 (7)		4 (5.6)		0.03	
high		5 (26.3)		3 (15.8)		9 (47.4)	2 (10.5)				8 (42.1)		5 (26.3)		2 (10.5)		4 (21.1)			
Serum ALT																				
low		0 (0)		34 (94.4)		2 (5.6)	0 (0)		0.48		22 (61.1)		8 (22.2)		3 (8.3)		3 (8.3)		0.989	
high		23 (42.6)		2 (3.7)		25 (46.3)	4 (7.4)				33 (61.1)		12 (22.2)		4 (7.4)		5 (9.3)			
Serum ferritin																				
low		20 (39.2)		19 (37.3)		11 (21.6)	1 (2)		0.001		39 (76.5)		7 (13.7)		2 (3.9)		3 (5.9)		0.001	
high		3 (7.7)		17 (46.3)		16 (41)	3 (7.7)				16 (41)		13 (33.3)		5 (12.8)		5 (12.8)			

*Fisher-exact test.

We compared the rates of high AST, ALT, and ferritin levels in different stages of liver and cardiac iron load with χ^2^ test. Neither high serum ALT nor AST were associated with liver iron load (*P* = 0.48 and 0.107, respectively) (Table 2). High AST but not ALT was associated with severe cardiac iron load (*P* = 0.03), as patients with high AST were 1.37 times more likely to have moderate to severe cardiac iron load (OR 1.38, 95% CI 1.013-2.11, *P* = 0.015).

High serum ferritin levels were associated with higher liver and cardiac iron load (*P* = 0.001 and 0.001, respectively) (Table 2). Patients with high serum ferritin were 2.068 times more likely to have high liver iron load (OR 2.068, 95%, 95% CI 1.26-3.37, *P* = 0.001). They were also 1.87 times more likely to have high cardiac iron load (OR 1.87, 95% CI 1.38-2.54, *P* = 0.001).

Patients with self-reported irregular use of iron chelating agents were more likely to have higher cardiac iron load (*P* = 0.028), but not liver iron load (*P* = 0.110). The type of iron chelators used was not associated with liver or cardiac iron load (Table 3).

**Table 3:** Liver and heart iron load according to regularity and type of iron-chelating agent (ICA)

		No. (%) of patients with																			
Parameter		liver iron load										heart iron load									
		normal		mild		moderate		severe		P*		normal		mild		moderate		severe		P*	
Use of ICA																				
regular	19 (31.1)		23 (37.7)		17 (27.9)		2 (3.3)		0.110		41 (67.2)		14 (23)		4 (6.6)		2 (3.3)		0.028	
irregular	4 (13.8)		13 (44.8)		10 (34.5)		2 (6.9)				14 (48.3)		6 (20.7)		3 (10.3)		6 (20.7)			
Type of ICA																				
deferoxamine (DFO)	11 (21.2)		20 (38.5)		18 (34.6)		3 (5.8)		0.222		30 (57.7)		9 (17.3)		6 (11.5)		7 (13.5)		0.092	
deferasirox (DFX)	9 (30)		12 (40)		8 (26.7)		1 (3.3)				22 (73.3)		7 (23.3)		1 (3.3)		0 (0)			
DFO and DFX	3 (37.5)		4 (50)		1 (12.5)		0 (0)				3 (37.5)		4 (50)		0 (0)		1 (12.5)			

*Fisher-exact test.

Significant variables from previous analyses were included in the multiple regression analysis. The significant predictor of liver iron load was serum ferritin (*R^2^* = 0.263, F(1, 88) = 6.51, *P* = 0.012). When cardiac iron load was entered into a regression model with serum AST, serum ferritin, and regularity of use of iron chelators, only serum ferritin was able to add predictive value to the model estimate, with an R^2^ = 0.314 (F (3, 86) = 3.77) and *P* = 0.014.

## Discussion

This study for the first time showed that higher serum ferritin strongly predicted the severity of hepatic and cardiac iron load. Irregular use of chelator drugs was associated with a higher risk of myocardial and liver iron load, regardless of the chelating agent type.

Over the last decade, life expectancy of patients with transfusion-dependent TM has dramatically improved. Cardiac complications, which can be divided into those caused by iron-overload and those not caused by non-iron-overload, are crucial predictors of the quality of life and mortality in these patients (29). Liver iron overload is an important indicator of iron balance status in patients with TM. Its robust correlation with whole body iron has made it a valuable tool that can be used when tailoring the iron chelators treatment (30).

A major problem in studies focusing on the efficacy of iron chelation therapy is to accurately evaluate patients’ compliance with DFO and DFX, which we calculated based on completed infusions and a self-report questionnaire, respectively. A combined treatment with DFO and DFX is more commonly prescribed in patients with high serum ferritin, AST, and/or ALT values at baseline, or as an urgent iron detoxification treatment when severe cardiovascular morbidities are present (31,32). Among our patients, those with the irregular use of iron chelating agents were more likely to have higher grades of iron load in cardiac tissue, but not in the liver; while cardiac or hepatic iron load was not significantly associated with the type of chelating agent. Therefore, the dosage and regularity of iron chelation had a greater impact on cardiovascular morbidity in these patients than the type of chelating drug.

LIC is the most accurate proxy of total body iron burden as more than 70% of total iron is stored in the liver (33). Moreover, hepatic siderosis correlates with iron overload in other organs. However the correlation with iron overload in the heart was observed with only in the cases of heavy iron deposition in the myocardium. This weak correlation might be explained by differences in iron kinetics, transferin receptor concentration, and the level of fibrosis or inflammation (34,35).

MRI-based organ assessment is now globally introduced as the gold standard for screening of liver and cardiac iron overload, replacing tissue biopsy and other invasive methods (36). The use of this method led to the improvement of both LIC and cardiac iron overload compared with the pre T2*MRI era (37). Nonetheless, Kolnagou et al (17) found that iron stores were not predicted by ferritin, but rather by chelation protocol, and genetic and dietary factors. This is in line with the findings that serum ferritin level was not a reliable predictor of liver iron load in patients with thalassemia intermedia under regular RBC transfusion (19).

We showed for the first time that ferritin can predict iron overload in the myocardium and liver tissues. Zamani et al (38) revealed no correlation between the histological grade of siderosis (HGS) and serum ferritin, but found a moderate correlation between serum ferritin levels and hepatic T2* levels. The correlation between ferritin and heart and liver T2*MRI values might be used as a proxy to estimate body iron levels.

Iron overload in patients with beta-TM is a direct result of transfusion, although it has also been associated with a few genetic loci (39). In patients with thalassemia intermedia, regardless of blood transfusion, the high risk of iron overload might be predicted by mutations in HFE gene (40).

The significance of total body iron status in tailoring chelation therapy and the selection of the most appropriate management strategies is under-appreciated. Several surveys have documented the impact of MRI availability on clinical outcomes of transfusion in patients with anemia (41,42). Also, appropriate iron chelation not only improves hematopoietic insufficiency in patients with myelodysplastic syndrome but also lowers the rate of progression toward leukemia, possibly by mitigating the mutagenic effect of iron free radicals (43).

Limitations of this study include a single assessment of AST, ALT, and serum ferritin based on laboratory data for each patient. Usually the average of the values obtained in the last 6 months is considered; however there are studies reporting a single measurement of serum ferritin values in relation with T2* MRI in beta- TM patients (18). Moreover, MRI iron measurements were read locally and were not centrally validated.

In conclusion, our results strongly support that iron and oral chelation in thalassemia patients should be assessed at least annually with T2*MRI. A consensus recommendation of previous studies was to obtain the first T2*MRI at the age of 10, with more frequent imaging if T2* is less than 10-20 ms. In transfusion-dependent TM patients, it is also recommended to annually measure hepatic markers, although it was not specified starting from what age, with the aim of adjusting these markers to chelation (44). Nonetheless, the prevention of iron overload in TM requires the clinicians’ cooperation with pathologists and biochemists. Future studies should investigate the ability of cost-effective serum markers to monitor iron overload in crucial organs.
